# A bibliometric and visual analysis based on immune checkpoint inhibitors for hepatocellular carcinoma: 2014 – 2024

**DOI:** 10.3389/fphar.2025.1520055

**Published:** 2025-04-07

**Authors:** Gao-Min Liu, Rui Guo, Ji-Wei Xu

**Affiliations:** ^1^ Meizhou Clinical Medical College of Shantou University Medical College, Meizhou, China; ^2^ Department of Hepatobiliary Surgery, Meizhou People’s Hospital, Meizhou, China

**Keywords:** immune checkpoint inhibitors, HCC, bibliometric, citespace, VOSviewer, R

## Abstract

**Background:**

Immune checkpoint inhibitors (ICIs) have changed the treatment landscape of hepatocellular carcinoma (HCC), especially those with unresectable advanced stages. The field has progressed rapidly, and the research hotspots have significantly changed compared to previous years. The study aims to comprehensively review and analyze the development history, knowledge structure, current research focus, and emerging trends in ICIs for HCC.

**Materials and methods:**

Reviews and articles published in English from The Web of Science Core Collection (WoSCC) database from 2014 to 2024 were systemically retrieved. Citespace, VOSviewer, and Bibliometrix R package were used for further bibliometric analysis and visualization for countries, institutions, authors, references, and keywords.

**Results:**

2,941 records were included for analysis. The literature on ICIs for HCC has continued to grow steadily over the past decade. Three major research centers have emerged: North America, Europe, and East Asia. The Chinese institution has the highest publication volume, but Kudo Masatoshi from Japan has the highest number of publications. At the same time, Richard S. Finn from the United States leads in citations and co-citations. The most prolific journal is “Cancers”. The clustering and Timeline view of critical literature and keywords indicated that research on ICIs for HCC is rapidly advancing toward a more evidence-based, personalized, and multimodal approach. Immune evasion mechanisms, predictive biomarkers, and high-quality clinical trials focusing on Novel combination, conversion, and perioperative therapies, including ICIs, are emerging hotspots.

**Conclusion:**

This study highlights the groundbreaking advancements of ICIs in treating HCC and shows a trend rapidly advancing towards a more evidence-based, personalized, and multimodal approach. The study updated the current understanding of ICIs in hepatocellular carcinoma and identified vital future directions for research, such as the exploration of mechanisms of immune evasion, developing predictive biomarkers, and combining therapy strategies.

## 1 Introduction

As the most common type of liver cancer, hepatocellular carcinoma (HCC) is still a significant global health challenge. In the past, HCC primarily developed in patients with chronic viral hepatitis or cirrhosis. With the control of viral hepatitis through vaccination and antiviral drugs, the burden of liver cancer is expected to decline significantly in endemic areas. However, the incidence of other risk factors, such as non-alcoholic fatty liver disease due to metabolic disorders, is rising. As a result, the incidence of HCC continues to increase in certain regions. It is currently the sixth most common malignant tumor worldwide and ranks third among the leading causes of cancer-related deaths (Bray et al., 2024). Due to the lack of specific symptoms in the early stages of HCC, most patients are already diagnosed at an advanced stage. This situation is attributed mainly to the liver’s remarkable regenerative capacity and difficulty detecting the disease in its early phases. For advanced HCC, potential curative treatments such as liver transplantation, surgical resection, and ablation often do not meet expectations and may frequently be unsuitable (Alessandro et al., 2023). Even those who receive curative treatment face a high risk of recurrence and metastasis (Carla et al., 2024). The treatment of HCC remains challenging, underscoring the urgent need for more effective options.

Over the past 20 years, targeted therapies like sorafenib and lenvatinib have progressed in advanced HCC (Kazufumi et al., 2023). However, there are significant challenges. These include low response rates, drug side effects, and increasing chemoresistance (Jin et al., 2021). These issues hinder both the success of treatment and its wider adoption. Immune checkpoint inhibitors (ICIs), a type of immunotherapy, have shown significant promise since the completion of the nivolumab Phase I/II clinical trial for advanced HCC in 2017 (El-Khoueiry et al., 2017). Subsequent research has continued to accumulate, highlighting their potential and vitality. ICIs block crucial immune checkpoint proteins hijacked by tumors, such as Cytotoxic T Lymphocyte Associated Protein 4 (CTLA-4), Programmed Cell Death Protein 1 (PD-1), and Programmed Cell Death Ligand 1 (PD-L1), therefore reversing tumor immune escape and enhancing the body’s immune response to tumors (Ke-Yu et al., 2024). Several ICIs have been approved for first-line or second-line treatment of advanced HCC, significantly improving patient prognosis (John et al., 2024). However, as research progresses, immunotherapy’s limitations and adverse effects are increasingly recognized. Topics such as combination therapies, conversion treatments, and predicting immune therapy responses are emerging as new research hotspots (Josep et al., 2024).

This study utilizes bibliometric methods to comprehensively review and analyze the development history, knowledge structure, current research focus, and emerging trends in ICIs for HCC. The goal is to provide valuable reference points for further research and practical applications in this field. There have been two previous bibliometric reviews of immunotherapy for HCC: one covering the period from 2011 to 2020 (Shen et al., 2022) and the other from 2021 to 2022 (Li et al., 2023). Notably, these reviews focused on overall immunotherapy rather than specifically on ICIs. ICI research has progressed rapidly, with a surge in relevant publications and significant shifts in research interests and hotspots (Tim et al., 2023; Parissa et al., 2024). Therefore, it is necessary to reassess the advancements and critical topics in this area.

## 2 Materials and methods

### 2.1 Data selection

**FIGURE 1:**
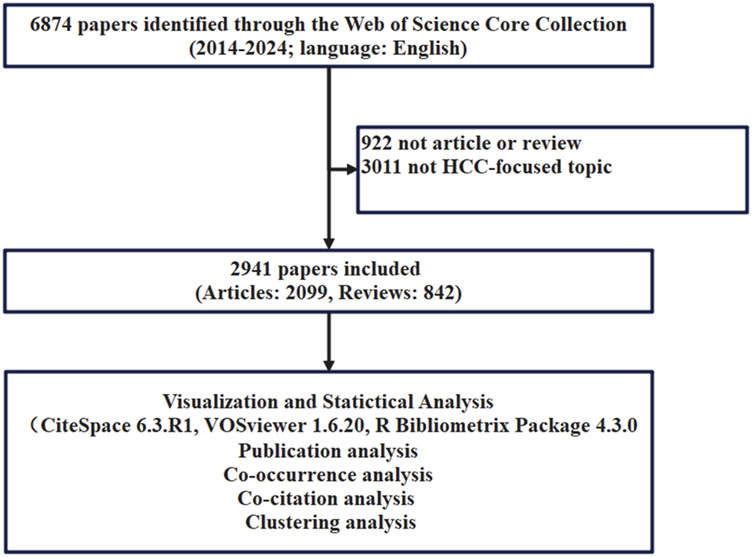
The flow chart of the study. HCC, hepatocellular carcinoma.

The input data for this study were obtained from the Web of Science Core Collection through a free word joint subject term search method. The terms “HCC” and “immune checkpoint inhibitors” were used. Our primary search terms included topic search (TS) = ((((liver OR hepatic OR hepatocellular OR hepato-cellular) AND (cancer* OR carcinom*OR neoplasm? OR maglinan* OR tumor* OR tumour*))) OR (HCC OR hepatoma?)), and TS = (“Immune Checkpoint Inhibitors” OR “ICIs” OR “CTLA 4 Inhibitors” OR “PD-1 Inhibitors” OR “PD-L1 Inhibitors” OR “Nivolumab” OR “Pembrolizumab” OR “Sintilimab” OR “Camrelizumab” OR “Tislelizumab” OR “Toripalimab” OR “Penpulimab” OR “Atezolizumab” OR “Durvalumab” OR “Envafolimab” OR “Ipilimumab” OR “Tremelimumab”). The detailed search strategy is described in Supplementary Table S1. Then, we restricted access to data from 2014 to 13 August 2024, limiting the literature to reviews and articles published in English to obtain 6,874 records. Then, we manually verified each retrieved article and found that many themes included literature related to other tumors, such as non-small cell lung cancer and melanoma. 3,933 studies unrelated to this research topic were identified and excluded from the final analysis. Finally, a total of 2,941 literature were included (Figure 1). All bibliographic information is downloaded and saved as Plain Text Files or Tab Delimited Files, and the records are complete records with cited references. Subsequently, the data were imported into Citespace 6.3. R1 (64-bit), VOSviewer (1.6.20), and Bibliometrix R package 4.3.0. During keyword co-occurrence analysis, synonyms were combined and processed.

### 2.2 Citespace (6.3.R1)

2,941 records in Plain Text File format were imported into CiteSpace for visual analysis, and the absence of duplicates was confirmed. Node type: “author, institution, country/region, keyword, reference, cited author, cited journal”; slice length:1 year; “Top N = 50″, g index (k = 2 or 5) was selected was selected. Pathfinder” and “Pruning sliced networks” were chosen to merge networks. Other parameters are set as default.

The larger the node, the more frequently the entry/reference appears, and the thicker the line indicates a stronger connection. The purple outer circle (centrality ≥1) indicates that the node has mediated centrality, i.e., central status, and is a major intellectual turning point. Red annual rings refer to bursts, indicating that the citation frequency is increasing rapidly. The visualization is colored from purple to red, indicating the average year from early to late (Fu et al., 2021; Xia et al., 2022).

Bursts reveal the evolution of research themes and can provide predictors of research frontiers and trends. Clustering can demonstrate disciplinary frontiers, i.e., emerging theoretical trends and the emergence of new topics. The clustering order starts from 0. The smaller the number, the more nouns/phrases are included. CiteSpace algorithmic clusters closely related nouns/phrases and assigns them a value, and the one with the most significant value in the same cluster is used as the representative of that cluster, i.e., it becomes a tag. When the clustering function begins, the Modularity Q and Mean Silhouette scores represent the overall structural characteristics of the network. Overall, Q > 0.3 indicates an overall significant structure. If S > 0.5, the clustering is usually considered reasonable, and S > 0.7 means the clustering is convincing (Chen, 2017; Chen; Chen et al., 2010).

### 2.3 VOSviewer (1.6.20)

2,941 records in Tab Delimited File format were retrieved from WoSCC and imported into VOSviewer to analyze collaborative networks (countries, institutions, authors, journals, keywords) and keyword co-occurrence. Keyword co-occurrence thresholds were set to ≥5 occurrences and link strength ≥2 to filter noise. The Full Counting method and LinLog/Modularity clustering (resolution = 1.0, minimum cluster size = 1) were applied to construct networks. Visualization parameters, including Attraction (Alessandro et al., 2023) and Repulsion (Bray et al., 2024), were optimized for layout clarity. Notably, data from China is divided into three regions (including mainland China, Hong Kong, and Taiwan) in WOS.

### 2.4 Bibliometrix R package 4.3.0

2,941 records in Plain Text File format were retrieved from the WoSCC database. Using the biblioshiny function of the Bibliometrix R package to analyze the country collaboration in the field. Briefly, the raw file of the retrieved records was imported into biblioshiny and processed automatically. Country collaboration networks were generated via the Countries’ Collaboration World Map of the social structure domain. The collaborative edge between different countries weights based on joint publication counts. All other parameters were defaulted.

## 3 Results

### 3.1 Scientometric analysis

#### 3.1.1 Summary of the documents

2,941 records, including 2099 articles and 842 reviews, were recognized. The average age of the records was 2.78 years. The average citations were 23.23 per record. The h-index was established at 105, indicating that 105 publications have been cited over 105 times, highlighting their importance and broad recognition within the field. 3,033 institutions from 81 countries/regions participated in the study, with contributions from 14,587 authors. These papers were disseminated across 559 different journals, utilizing a total of 5,659 keywords, which included 3,527 author keywords.

#### 3.1.2 Number of annual publications

**FIGURE 2:**
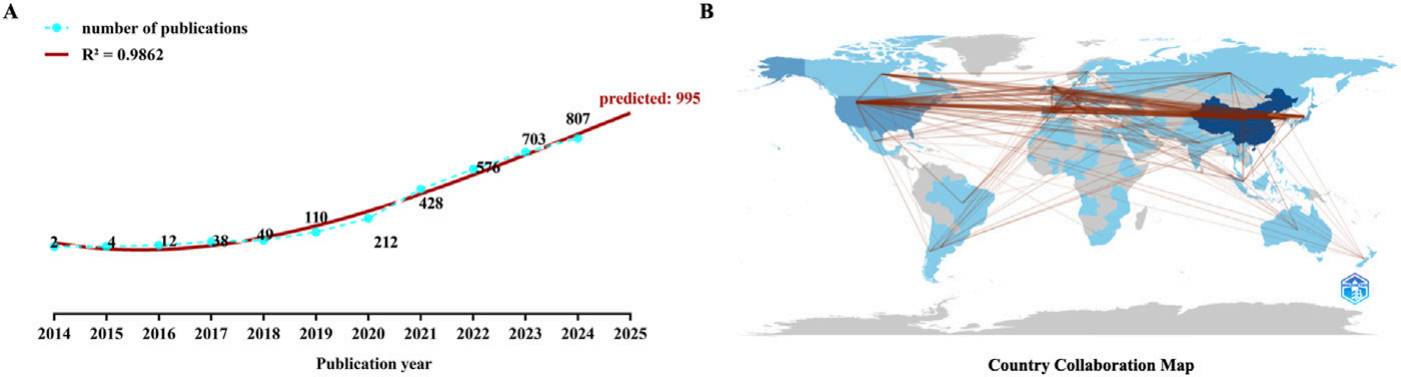
Numbers and country distribution of the related publications **(A)** The polynomial curve fitting of publications in immune checkpoint inhibitors for hepatocellular carcinoma **(B)** Analysis of countries’ distribution of the related publications in this field.

Figure 2A shows the annual publication volume, highlighting a steady increase from 2014 to 2024. A polynomial regression model was used to fit the yearly publication volume and predict that the annual output 2025 may reach 995. The publication volume for 2023-2024 exceeded the combined total for 2014-2022, indicating a sustained increase in research interest and data accumulation.

#### 3.1.3 Geographical disparity of the publication

Figure 2B shows the distribution of source countries for the relevant literature. It can be observed that the research primarily formed three centers: North America, Europe, and East Asia.

### 3.2 Related countries/regions and institutions analysis

#### 3.2.1 Related countries/regions analysis

**FIGURE 3:**
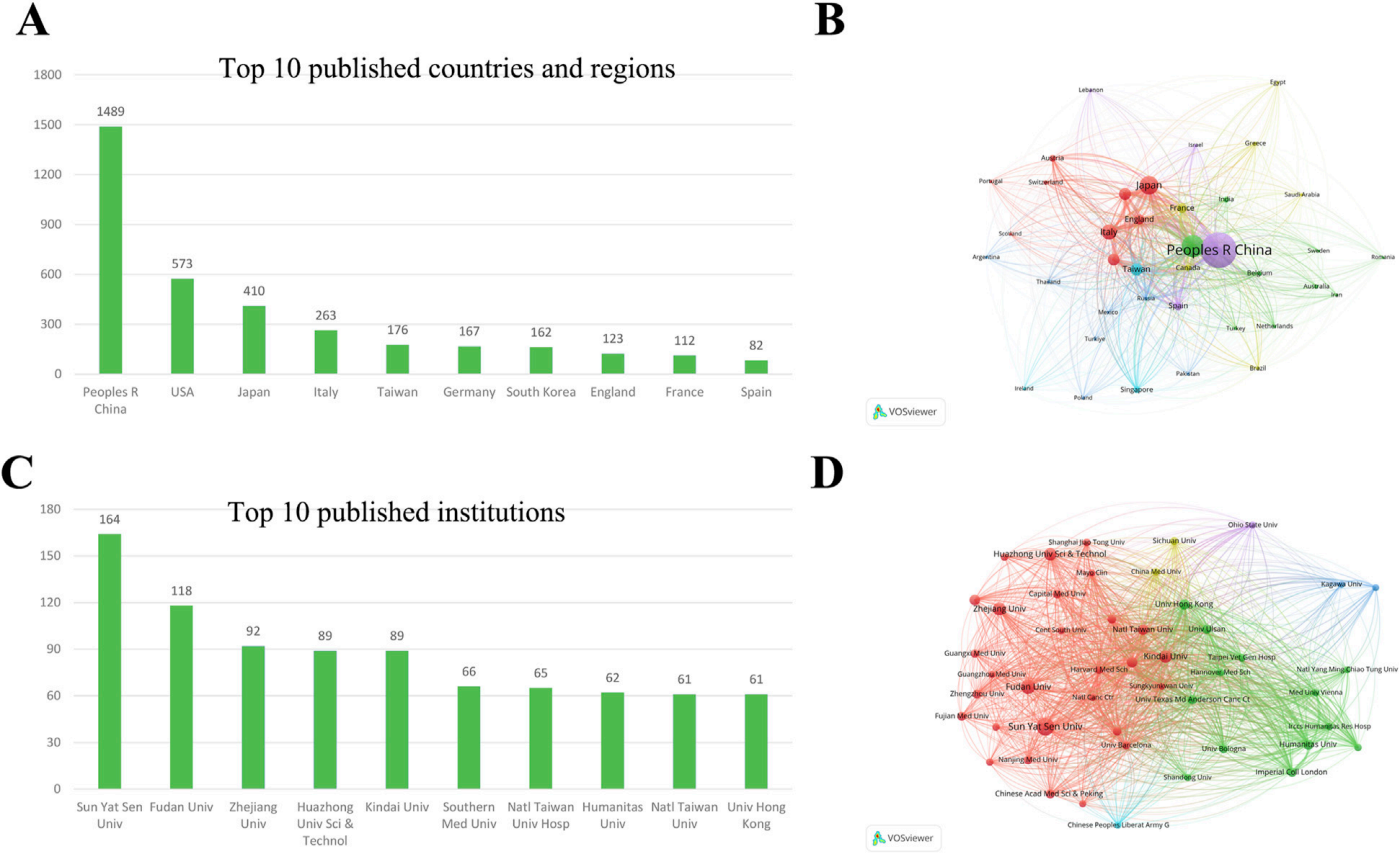
Visualization of countries and institutions’ co-occurrence networks in ICIs for HCC **(A)** The top 10 most productive countries and regions **(B)** Visualization of country/region co-occurrence network **(C)** The top 10 most productive institutions **(D)** Visualization of institution co-occurrence network. ICIs, immune checkpoint inhibitors; HCC, hepatocellular carcinoma.

VOSviewer was used to analyze the country’s and region’s distribution. Figure 3A shows the top 10 published countries and regions. Twenty-seven countries or areas have published more than ten papers in this field (Figure 3B). Among them, China published the most literature (1,489, 50.63%), followed by the United States (573, 19.48%) and Japan (410, 13.94%).

#### 3.2.2 Related institutions analysis

All authors of the 2,941 sample articles originated from 3,033 institutions, indicating the wide range of institutions dedicated to ICIs and HCC research. Figure 3C presents the top 10 institutions with the highest number of publications. Sun Yat-sen University leads with 164 articles, constituting 5.58% of all published papers. Fudan University follows this with 118 publications (4.09%) and Zhejiang University with 92 (3.00%). Among the top 10 institutions, five are from Mainland China, two from Taiwan, one from Hong Kong, one from Japan, and one from Italy. 90 institutions have published over 20 papers in this field (Figure 3D).

### 3.3 Author analysis

#### 3.3.1 Most productive authors

**FIGURE 4:**
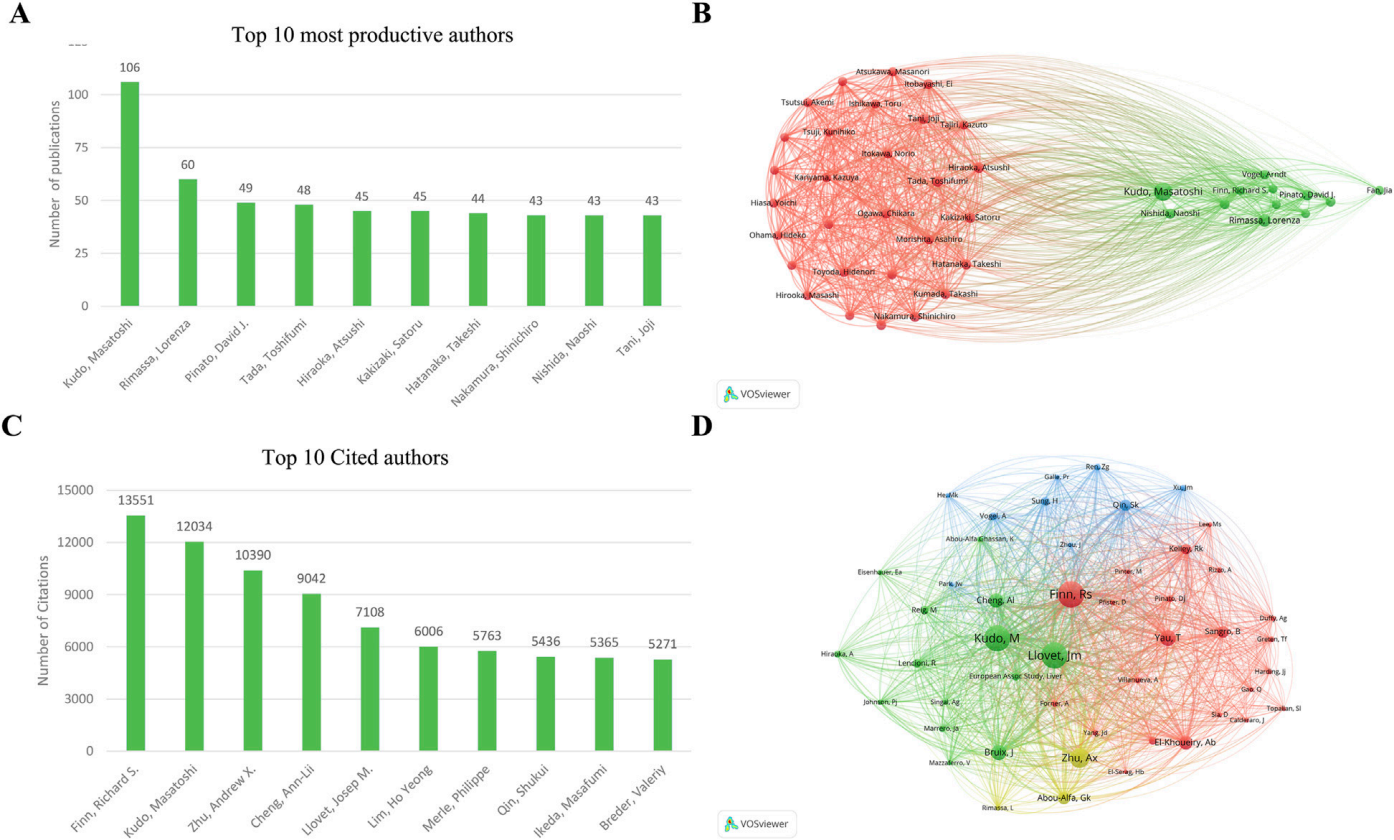
Visualization of authors and cited authors’ co-occurrence networks in ICIs for HCC **(A)** The top 10 most productive authors **(B)** Visualization of author co-occurrence network **(C)** The top 10 most cited authors **(D)** Visualization of cited author co-occurrence network. ICIs, immune checkpoint inhibitors; HCC, hepatocellular carcinoma.

VOSviewer analysis showed that 14,587 authors have participated in studies focusing on ICIs for HCC. Among them, 84 authors have published over 20 papers in this field. The top three prolific authors are Kudo and Masatoshi, with 106 papers. This is followed by Rimassa, Lorenza, Pinato, and David J, each with 60 and 49 papers (Figures 4A, B). Finn, Richard S had the most citations, with 13,551 citations. This is followed by Kudo, Masatoshi, and Andrew X. Zhu, each with 12,034 and 10,390 citations (Figure 4C). Notably, Finn, Richard S, and Kudo, Masatoshi, authored the two most frequently cited papers.

#### 3.3.2 Co-citation analysis

Figure 4D shows the functional and thematic influence of authors of authors who have cited over 210 papers. The analysis generated an interconnected network of 48 authors. In the graph, each node signifies an author, where the size of the circle corresponds to the number of articles published by that author, and transparency increases as the article count decreases. The lines connecting the circles vary in number and thickness, representing the co-occurrence relationships among authors. Authors of the same color belong to the same cluster, suggesting their research is frequently co-cited.

Finn, Richard S leads with 3,327 co-citations among the top three co-cited authors. He is followed by Kudo, Masatoshi, with 3,268 co-citations, and Llovet, Josep M., with 3,261 co-citations. Several prominent co-citation relationships exist, such as 1) Josep M. Llovet and Masatoshi Kudo and 2) Andrew X. Zhu and Ann-Lii.

### 3.4 Journals

#### 3.4.1 Most productive journals

**FIGURE 5:**
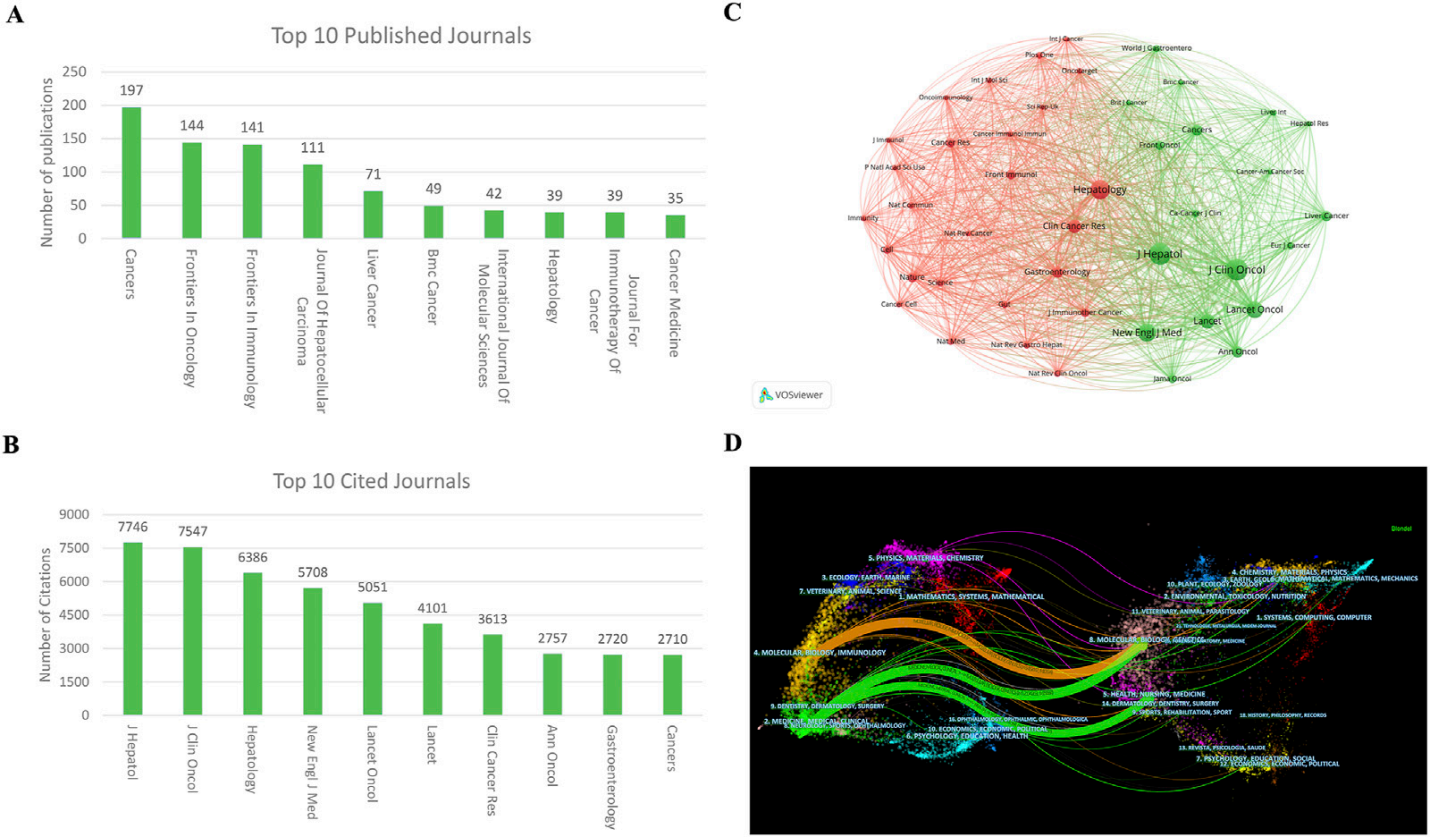
Visualization of journals and cited journal co-occurrence networks in ICIs for HCC **(A)** The top 10 most productive journals **(B)** Visualization of journal co-occurrence network **(C)** The top 10 most cited journals **(D)** Dual map showing the overlay of journal themes. ICIs, immune checkpoint inhibitors; HCC, hepatocellular carcinoma.

A total of 559 journals have published articles concerning ICIs for HCC. The journal “Cancers” (impact factor 4.5 for 2023) published the most articles with 197 articles, followed by “Frontier in Oncology” (impact factor 3.5 for 2023) with 144 articles and “Frontiers in Immunology” (impact factor 5.7 for 2023) with 141 articles. (Figure 5A). Among the top 10 cited journals in this field, the “Journal of Hepatology” (impact factor 26.8 for 2023) holds the top rank with 7,746 citations and 33 articles, followed by the “Journal of Clinical Oncology” (impact factor 32.956 for 2023) with 7,547 citations and seven articles and “Hepatology” (impact factor 12.9 for 2023) with 6,386 citations and 39 articles (Figure 5B). Figure 5C illustrates the positive citation relationships among these journals.

#### 3.4.2 Dual map overlay analysis

Figure 5D presents a dual map overlay illustrating journal themes. Journals that cite articles are depicted on the left side of the map, while the cited journals appear on the right side. The labels near the emitting area indicate their respective disciplines, with each label positioned around the cluster centroid of the corresponding journals. Colored lines extending from left to right represent the citation pathways. A longer vertical axis of the ellipse signifies more published papers, while a longer horizontal axis indicates a more significant number of authors associated with the journal. There are four primary citation pathways. One orange pathway indicates that molecular/biology/immunology journals often reference journals in molecular/biology/genetics. Meanwhile, the two green paths show that research journals from the molecular/biology/genetics and health/nursing/medicine areas are frequently cited in medicine/medical/clinical journals.

### 3.5 Literature analysis

#### 3.5.1 Most cited literatures

Table 1 describes the top 10 kinds of literature involved in ICIs for HCC. Among these studies, the IMbrave150 research titled “Atezolizumab plus Bevacizumab in Unresectable Hepatocellular Carcinoma” (Finn et al., 2020a), authored by Richard S. Finn et al. and published in the “New England Journal of Medicine” in 2020, has been cited the most (n = 3,834). This research has significantly affected the treatment landscape for ICIs in HCC and has sparked extensive discussion and subsequent follow-up studies.

**TABLE 1:** The top 10 literatures involved in immune inhibitor inhibitors research in relation to HCC.

Reference	Count	DOI
Finn RS, 2020, NEW ENGL J MED, V382, P1894	3,834	https://doi.org/10.1056/nejmoa1915745
Llovet JM, 2021, NAT REV DIS PRIMERS, V7, P0	2,504	https://doi.org/10.1038/s41572-020-00240-3
Yang JD, 2019, NAT REV GASTRO HEPAT, V16, P589	2,476	https://doi.org/10.1038/s41575-019-0186-y
Zhu AX, 2018, LANCET ONCOL, V19, P940	1722	https://doi.org/10.1016/s1470-2045(18)30351-6
Zheng CH, 2017, CELL, V169, P1342	1,345	https://doi.org/10.1016/j.cell.2017.05.035
Llovet JM, 2018, NAT REV CLIN ONCOL, V15, P599	1,248	https://doi.org/10.1038/s41571-018-0073-4
Finn RS, 2020, J CLIN ONCOL, V38, P193	1,209	https://doi.org/10.1200/jco.19.01307
Yau T, 2020, JAMA ONCOL, V6, P0	787	https://doi.org/10.1001/jamaoncol.2020.4564
Llovet JM, 2022, NAT REV CLIN ONCOL, V19, P151	758	https://doi.org/10.1038/s41571-021-00573-2
Finn RS, 2020, J CLIN ONCOL, V38, P2960	710	https://doi.org/10.1200/jco.20.00808

#### 3.5.2 Co-citation analysis

**FIGURE 6:**
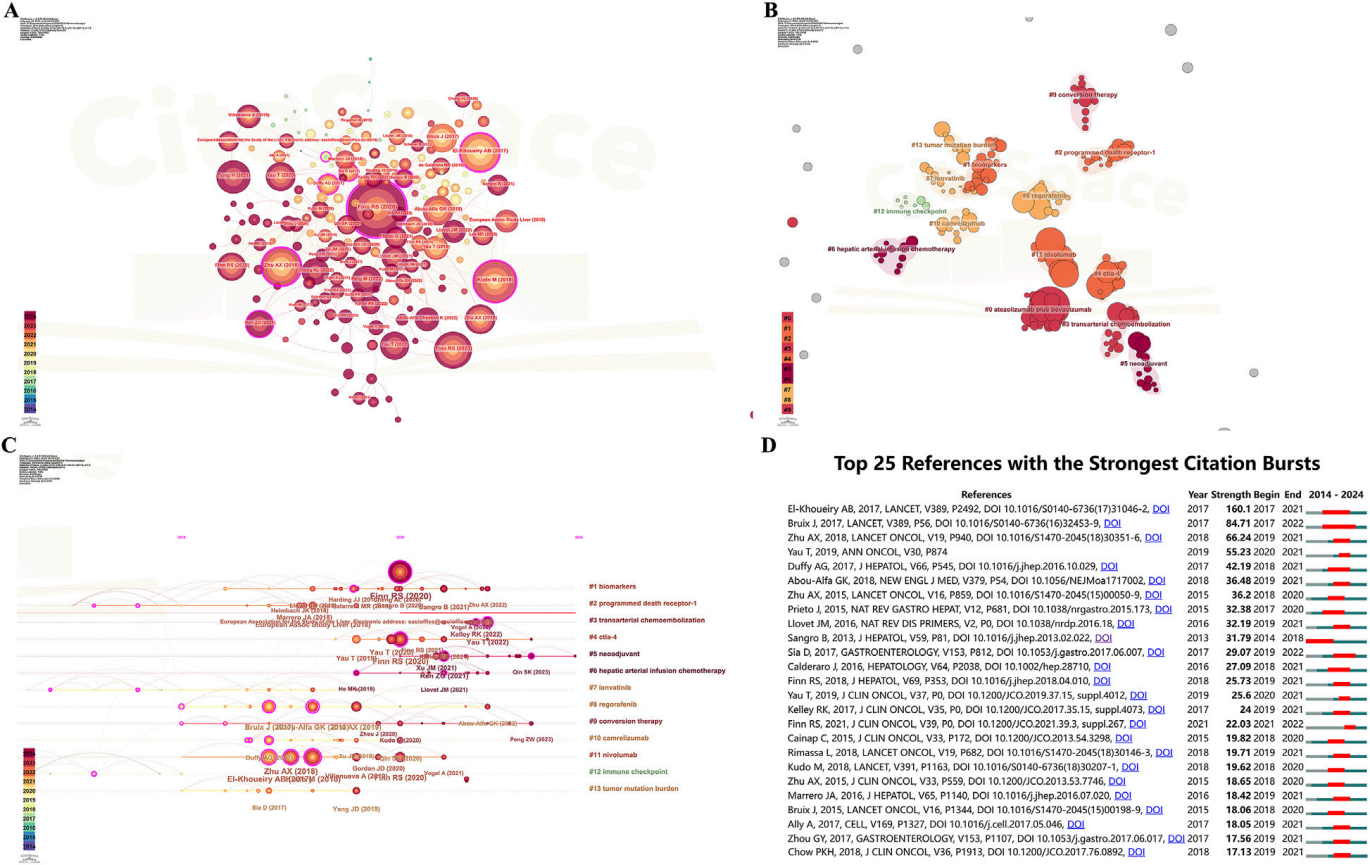
Visualization of literature networks in ICIs for HCC **(A)** Co-citation network of the literature **(B)** Visualization of literature clustering network **(C)** The timeline view of the literature clustering **(D)** The top 25 references with strongest citation burst. ICIs, immune checkpoint inhibitors; HCC, hepatocellular carcinoma.

CiteSpace was used to generate a co-citation network of the literature to uncover the progression of ICIs for HCC (Figure 6A). Most highly cited literature was published in the last 5 years. The topmost explosive documents were highlighted with labels.

#### 3.5.3 Cited literature clustering analysis

CiteSpace was used to generate a clustering graph of the cited literature, select keywords as the marker source, and use automatic clustering to obtain 13 significant clusters. This visualization plot Q = 0.8736, S = 0.9693, and the clustering results are convincing. As shown in Figure 6B, the first three clusters are labeled as “#0 atezolizumab plus bevacizumab”, “#1 biomarkers”, and “#2 programmed death receptor-1”, respectively.

#### 3.5.4 Timeline view

The timeline view of the literature clustering illustrates nodes representing individual references, where larger nodes signify a more significant number of citations. Nodes on the left represent earlier references, while those on the right correspond to more recent ones. Nodes positioned along the same line create a cluster, each labeled with a number (#) on the right side.

The timeline view indicated several emerging research hotspots, including #0 programmed death receptor-1, #1 biomarkers, #2 programmed death receptor-1, #3 transarterial chemoembolization, #4 ctla-4, #5 neoadjuvant, #6 hepatic arterial infusion chemotherapy, #7 lenvatinib, #8 regorafenib, #9 conversion therapy, #10 camrelizumab, #11 nivolumab, #12 immune checkpoint, and #13 tumor mutation burden (Figure 6C).

#### 3.5.5 Literature citation burst

Figure 6D shows the top 25 references with the most robust citation burst. The green line indicates the timeframe from 2014 to 2024, while the red line represents the duration of each citation burst. The minimum burst duration was set as 2 years. The literature with the highest mutation intensity is the paper by El-Khoueiry AB et al. titled “Nivolumab in patients with advanced hepatocellular carcinoma (CheckMate 040): an open-label, non-comparative, phase 1/2 dose escalation, and expansion trial.”

### 3.6 Keyword analysis

#### 3.6.1 Keyword co-occurrence

**FIGURE 7:**
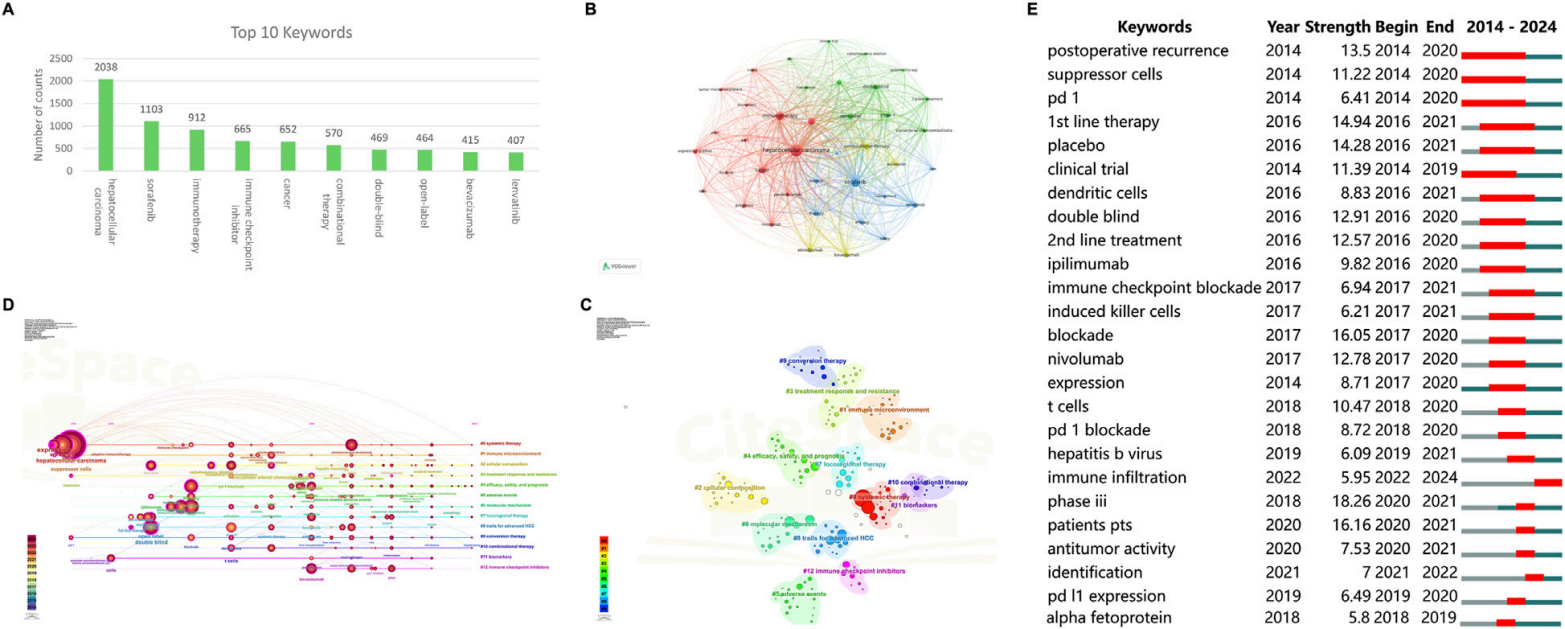
Visualization of author keyword networks in ICIs for HCC **(A)** The top 10 most frequently occurring keywords **(B)** Visualization of keyword co-occurrence network **(C)** Visualization of keyword clustering network **(D)** The timeline view of the keyword clustering **(E)** The top 25 keywords with strongest citation burst. ICIs, immune checkpoint inhibitors; HCC, hepatocellular carcinoma.

Synonymous keywords were merged before analysis. Since this study focused on HCC and ICIs, these two keywords were first excluded when high-frequency keywords were selected. As shown in Figure 7A, the most frequently occurring keywords, in order of frequency, are sorafenib, immunotherapy, cancer, combinational therapy, double-blind, open-label, bevacizumab, and Lenvatinib. Figure 7B illustrates a co-occurrence map that visualizes the relationships between keywords. The keywords encompass three main themes: treatment strategies and outcomes for advanced HCC, the tumor immune microenvironment and biomarkers, and the application of ICIs in HCC. For instance, in the context of the tumor immune microenvironment and biomarkers, there is often a concurrent emphasis on predictive factors such as the tumor microenvironment and other biomarkers like immune checkpoint expression.

#### 3.6.2 Clustering and timeline view

CiteSpace was used to generate a clustering graph of all keywords and use automatic clustering to obtain 15 significant clusters. This visualization plot Q = 0.8226, S = 0.9718, and the clustering results are convincing. As shown in Figure 7C, the clusters included #0 systemic therapy, #1 immune microenvironment, #2 cellular composition, #3 treatment response and resistance, #4 efficacy, safety, and prognosis, #5 adverse events, #6 molecular mechanism, #7 locoregional therapy, #8 trails for advanced HCC, #9 conversion therapy, #10 combinational therapy, #11 biomarkers, #12 immune checkpoint inhibitors. Figure 7D shows the timeline view of the clusters. The keywords under each cluster are arranged according to the time of first appearance, and it should be noted that the large circle does not mean that the keyword appeared most frequently in that year but that the keyword was first cited in that year and then the citation frequency was cumulated in that year. The number of nodes on the timeline can reflect the cooling or heating of the research field in a given year. The timeline clusters could also be classified into three main themes: 1) treatment strategies and outcomes for advanced HCC (#0, #4, #5, #7, #8, #9, #10, #11); 2) the tumor immune microenvironment and biomarkers (#1, #2, #6); and 3) the application of ICIs in HCC (#12). In addition, the analysis indicated that the research interest in clusters #2, #3, #4, #6, #7, #9, and #10 has consistently risen. These studies suggest that as the efficacy and safety of ICIs (immune checkpoint inhibitors) in HCC (hepatocellular carcinoma) gain recognition, there is a growing enthusiasm for exploring combination therapies and conversion treatments, further expanding the application scenarios for ICIs.

#### 3.6.3 Keyword burst

Keyword bursting can be analyzed as a hot spot and frontier of research. These keywords were organized based on duration, start time, and burst intensity. “Postoperative recurrence” emerged as the earliest burst keyword (initial burst in 2014) with the longest sustained duration of 7 years (2014-2020), indicating that during that period, the in-depth research on postoperative recurrence and metastasis of HCC was one of the main reasons and enduring challenges that drove the further exploration of ICIs in HCC. “Phase III” has the highest burst intensity, indicating that critical phase III studies are a huge boost to the field of research (Figure 7E). The evolution of keywords from early “clinical trial” to discussions on “first- and second-line treatment” and then to combined local approaches, such as “hepatic resection” for unresectable HCC, indicates the ongoing development and maturation of ICI therapy for HCC, as well as a more specialized focus in research. Additionally, the bursts of keywords such as “dendritic cells,” “induced killer cells,” and “immune infiltration” highlight the increasing recognition of the critical role of the tumor immune microenvironment in ICI treatment.

## 4 Discussion

### 4.1 ICIs for HCC

The efficacy of traditional HCC treatments is gradually becoming bottlenecked. At the forefront of anti-tumor therapy, ICIs have become one of the primary therapies of advanced HCC and have shown great potential in the neoadjuvant/adjuvant and conversion setting (Enrui et al., 2023). ICI treatment tends to reverse tumor immune evasion, rebuild the body’s anti-tumor immunity, activate the body’s recognition and elimination of tumor cells, and achieve lasting killing of tumor cells. However, as the clinical application of ICIs deepens, many treatment-related challenges present themselves, and the research trends are significantly different from the beginning (Matthias et al., 2023; Ciro et al., 2023).

### 4.2 Bibliometric results

#### 4.2.1 Publication volume and trend

The literature on ICIs for HCC has continued to grow steadily over the past decade, with a predicted output of 957 articles in 2024. The publication volume for 2023-2024 is expected to surpass the combined total for 2014-2024. A diverse and expanding number of authors from various countries and institutions contribute to HCC research involving ICIs, rapidly propelling the field. Literature citations in related fields are very active. These results indicate a sustained increase in research interest and data accumulation. In the foreseeable future, research in ICIs for HCC will remain an absolute hotspot for HCC treatment. However, significant knowledge gaps persist regarding the potential for improved therapeutic agents, combinations of ICIs, and more individualized treatment approaches to enhance the prognosis of patients with hepatocellular carcinoma.

#### 4.2.2 Countries/regions and institutions contribution

Three major research centers have emerged: North America, Europe, and East Asia. China, the United States, and Japan published the most significant articles. Research universities or institutions, such as Sun Yat-sen University in China, dominate the top ten institutions. As the country with the highest incidence of liver cancer in the world, China faces a considerable health burden of hepatocellular carcinoma (HCC) and an urgent need for improved treatment; in contrast, developed countries such as the United States, Japan, and Europe benefit from well-established healthcare systems, adequate funding, and a strong capacity for innovation. Together, these factors have contributed to the development of ICI research. However, significant gaps remain in using ICI for HCC in regions with high HCC incidence, such as Southeast Asia and northern and western Africa. Even in represented regions, socioeconomic disparities have led to uneven application of ICIs, which cannot be overlooked (Zhao et al., 2024; Bteich et al., 2024). Regional analyses should be conducted to identify barriers to ICI adoption in underserved regions, with targeted solutions proposed, such as resource-sharing initiatives or region-specific clinical trials. Regional cost-effectiveness analyses are needed to guide healthcare policy development and proper treatment costs for ICIs (Mohammadnezhad et al., 2024). Furthermore, these regions may require more practical conversion strategies, affordable ICIs, and biomarkers than developed areas. To address these challenges, implementing cost-effective pricing mechanisms for ICIs, simplifying conversion strategies, and developing accessible biomarkers such as blood test-based biomarkers could significantly improve the adoption and effectiveness of ICIs in these regions. Besides, global collaboration and knowledge exchange are critical for advancing research and promoting healthcare equity across diverse areas.

#### 4.2.3 Author contribution

The Chinese institution with the highest publication volume has yet to produce the most prolific authors. In the author analysis, Kudo Masatoshi, who has the highest number of publications, is from Japan, while Richard S. Finn from the United States leads in citations and co-citations. Richard S. Finn and others published the landmark IMbrave150 research (Finn et al., 2020a), which groundbreakingly demonstrated that Atezolizumab plus Bevacizumab is more effective than sorafenib in the treatment of unresectable hepatocellular carcinoma, significantly advancing the field. Kudo Masatoshi is one of the authors of the milestone CheckMate 040 study (El-Khoueiry et al., 2017), which was the first to confirm the safety and efficacy of the PD-1 inhibitor Nivolumab in advanced HCC. This rapidly led to the inclusion of the drug in liver cancer treatment guidelines. Furthermore, Kudo Masatoshi has conducted in-depth explorations regarding adverse reactions and combination therapies in the context of ICIs for HCC (Ciro et al., 2023; Melero et al., 2024).

#### 4.2.4 Journal distribution

The most prolific journals publishing literature on ICIs and HCC include “Cancers,” “Frontiers in Oncology,” and “Frontiers in Immunology”. However, higher-quality articles are more commonly found in journals such as the “New England Journal of Medicine” and the “Journal of Hepatology.” High-quality journals predominantly publish groundbreaking research or comprehensive reviews that summarize current research advancements and broaden the scope of the research field (Donne and Lujambio, 2023; Faivre et al., 2020).

#### 4.2.5 Key literatures and keywords

In 2017, with the FDA’s approval of the “PD-1 inhibitor” Nivolumab for second-line treatment of advanced liver cancer, the field officially entered the era of immunotherapy (El-Khoueiry et al., 2017). As immunotherapy improves by leaps and bounds, mounting studies have found that the effectiveness of immunotherapy as monotherapy is insufficient, prompting a shift in medical strategies toward combination therapies. In addition to the emergence of new immune checkpoint inhibitors (ICIs) (such as pembrolizumab [26, 27], “ocrelizumab” (Qin et al., 2023a), “tremelimumab” (Abou-Alfa et al., 2022; Lau et al., 2024), and ipilimumab (Melero et al., 2024)), there is a growing number of combination therapy strategies (including ICIs (Patel et al., 2024), tyrosine kinase inhibitors (Finn et al., 2020b), and VEGF inhibitors (Ren et al., 2021)) and locoregional therapies (such as TACE (Lencioni et al., 2024), HAIC (Zhang TQ. et al., 2023), and radiotherapy (Zhu et al., 2024)) being utilized for first- and second-line treatment of hepatocellular carcinoma (HCC) (Cappuyns et al., 2024; China NHCotPsRo, 2024). However, such research is notably lacking for later-line therapies after the failure of first- and second-line regimens.

Treatment intolerance and resistance are the two main obstacles for patients to persist in using ICIs. A recent meta-analysis reported a 61.0% overall incidence of any-grade irAEs and 13.2% grade ≥3 immune-related adverse events (irAEs). The most common irAE of any grade is reactive cutaneous capillary endothelial proliferation, while the most common grade 3 or higher irAE is an elevation in aspartate aminotransferase (Wang et al., 2024). Cautious management should be highlighted, especially in patients receiving combination therapy or prior chemotherapy and patients with autoimmune diseases. Approximately 46.2% of HCC patients experience primary resistance; around 28.4% show secondary resistance to ICIs (Cui et al., 2024). The underlying mechanisms might be genetic and epigenetic factors such as “tumor mutation burden,” tumor microenvironment such as “immune infiltration,” and “adaptive immunity” (Zheng et al., 2017; Shen KY. et al., 2024; Ma J. et al., 2024; Xie et al., 2024). In addition, significant effort has been made to investigate the potential biomarkers panel that predict ICI efficacy and resistance (Shen YC. et al., 2024; Celsa et al., 2024a). Therefore, future research should focus on integrating multi-omics analyses and clinical translation to establish predictive models and develop precision intervention strategies to overcome ICI resistance while improving immune tolerance management in HCC patients.

ICI has revolutionized the treatment paradigm for initially unresectable hepatocellular carcinoma, as named conversion therapy (Deng et al., 2024). Conversion therapy has allowed patients to undergo complete surgical resection, helping to convert unresectable tumors into resectable ones. Nevertheless, some key issues remain to be resolved: lack of high-level evidence, no standardized inclusion criteria (Xu et al., 2024), uncertainty about the optimal conversion therapy (Zhang W. et al., 2023; Zhu et al., 2021), and the selection of the best timing for surgery. The choice between surgical resection (Ma Z. et al., 2024; Liu et al., 2024; Shimose et al., 2023) and a “watch-and-wait” strategy (Li et al., 2024) following conversion therapy remains contentious; however, emerging evidence suggests that achieving a complete pathological response may serve as a crucial predictor for evaluating the long-term efficacy of subsequent surgical interventions in conversion therapy regimens (Jiang et al., 2024; Zeng et al., 2024).

Moreover, the therapeutic integration of ICIs with neoadjuvant/adjuvant approaches in initially resectable HCC represents a critical knowledge gap requiring urgent investigation. Similar to conversion therapy research, the current evidence base primarily derives from retrospective analyses and small-scale prospective studies with inherent methodological constraints (Zhou et al., 2023). Notably, several landmark phase III trials are currently underway that warrant particular attention: The IMbrave 050 trial (NCT04102098) is prospectively comparing atezolizumab plus bevacizumab against active surveillance (Qin et al., 2023b), while the SHR-1210-III-325 study (NCT03821935) is evaluating camrelizumab combined with apatinib *versus* surveillance strategies.

Overall, from a timeline perspective, these results reflect active exploration and immense potential for ICIs in strategies aimed at achieving complete tumor resection. Together with the keyword “network meta-analysis” (Rezaee-Zavareh et al., 2024; Celsa et al., 2024b), research on ICIs for HCC is rapidly advancing towards a more evidence-based, personalized, and multimodal approach.

### 4.3 Current status and future perspectives

#### 4.3.1 Current status

Applying ICIs in HCC represents a significant advancement in the treatment landscape. ICIs have emerged as cornerstone treatments in advanced HCC, with their efficacy underpinning a shift in treatment goals. The focus has evolved from merely controlling the disease to achieving substantial or complete pathological responses, thereby enabling the potential for surgical resection in previously unresectable cases. Research on ICIs for HCC has steadily increased, with significant contributions from institutions in North America, Europe, and East Asia. Leading institutions have been pivotal in advancing research, but disparities remain, particularly in regions with high HCC incidences, such as Southeast Asia and parts of Africa. Despite notable advances with drugs like Nivolumab and Atezolizumab, challenges remain, such as limited objective response rates when used as monotherapy. Ongoing studies are exploring combination therapies and the mechanisms of ICI resistance while seeking predictive biomarkers to enhance treatment personalization. However, there is still a notable need for large-scale clinical trial evidence, particularly concerning the efficacy of ICIs after the failure of initial treatment regimens.

#### 4.3.2 Future direction

The future of ICI therapy in HCC holds several vital opportunities. Continued exploration of the immune evasion mechanisms in HCC will aid in developing new ICI drugs, understanding the mechanisms of immunotherapeutic resistance, and improving immunotherapy efficacy. Developing and validating predictive biomarkers, especially using artificial intelligence (AI)-driven biomarker discovery platforms, for immunotherapy efficacy will facilitate the formulation of individualized immunotherapy strategies. Exploring combination therapy strategies, including ICIs with novel systemic and locoregional treatments, is essential for improving patient outcomes. High-quality evidence from well-designed clinical trials will help shape treatment guidelines, particularly in later-line, conversion therapy, and perioperative settings. Global collaboration will address disparities in treatment access and share knowledge across high-incidence regions.

### 4.4 Advantages and limitations

This study conducted a systematic review focusing on the progress of ICIs in treating HCC. Through a meticulous and high-quality literature search, we provided a multi-dimensional analysis based on bibliometrics and an objective summary, updating current knowledge in this field and highlighting critical future directions. Nevertheless, the manuscript has some limitations. First, our study was limited to English literature indexed in the Web of Science Core Collection database, which means that literature from other databases like PubMed and Scopus and non-English sources was not included in the analysis. We exclusively used WOSCC because it focuses more on high-quality, high-impact research, while Scopus or other databased includes many conference proceedings and non-English sources outside our inclusion criteria. This approach might help improve analytical efficiency and ensure accurate identification of current hotspots and trends. Additionally, numerous classic bibliometric studies have prioritized WOSCC, facilitating direct comparisons with prior analyses in this field that also exclusively utilized WOSCC. Nevertheless, some key research might be lacking due to database constraints, language limitations, and delayed citation in the Web of Science Core Collection database. Future studies should expand data sources by including additional databases to enhance the comprehensiveness of the bibliometric analysis. Second, the inherent limitations of the bibliometric methodology and the research time frame resulted in significant shortcomings in assessing the breakthrough impact of very recent important literature and the contributions of multiple authors.

## 5 Conclusion

In summary, this study highlights the groundbreaking advancements of ICIs in treating HCC and shows a trend rapidly advancing towards a more evidence-based, personalized, and multimodal approach. The study updated the current understanding of ICIs in hepatocellular carcinoma and identified vital future directions for research, such as the exploration of mechanisms of immune evasion, developing predictive biomarkers, and combining therapy strategies. High-quality evidence and global collaboration will help address these challenges and share knowledge across high-incidence regions.

## Data Availability

The original contributions presented in the study are included in the article/Supplementary Material, further inquiries can be directed to the corresponding authors.
