# Supplementary material for: A bibliometric and visual analysis based on immune checkpoint inhibitors for hepatocellular carcinoma: 2014 – 2024
Source: Front Pharmacol. 2025 Apr 7;16:1520055. doi: 10.3389/fphar.2025.1520055 (PMC12009821; doi:10.3389/fphar.2025.1520055)
Supplement: Supplementary file 1 [file Table1.DOCX]

| **Table S1 Search strategy** | |
| --- | --- |
| **step** | **search or restrict word** |
| #1 | (“Immune Checkpoint Inhibitors“ or ICIs or ”Checkpoint Inhibitors, Immune“ or ”Immune Checkpoint Blockers“ or “Checkpoint Blockers, Immune” or “Immune Checkpoint Inhibitor” or “Checkpoint Inhibitor, Immune” or “CTLA-4 Inhibitors” or “CTLA 4 Inhibitors” or “Cytotoxic T-Lymphocyte-Associated Protein 4 Inhibitors” or “Cytotoxic T Lymphocyte Associated Protein 4 Inhibitors” or “Cytotoxic T-Lymphocyte-Associated Protein 4 Inhibitor” or “Cytotoxic T Lymphocyte Associated Protein 4 Inhibitor” or “CTLA-4 Inhibitor” or “CTLA 4 Inhibitor” or “PD-1 Inhibitors” or “PD 1 Inhibitors” or “Programmed Cell Death Protein 1 Inhibitor” or “Programmed Cell Death Protein 1 Inhibitors” or “PD-1 Inhibitor” or “Inhibitor, PD-1” or “PD 1 Inhibitor” or “Immune Checkpoint Blockade” or “Checkpoint Blockade, Immune” or “Immune Checkpoint Inhibition” or “Checkpoint Inhibition, Immune” or “PD-L1 Inhibitors” or “PD L1 Inhibitors” or “Programmed Death-Ligand 1 Inhibitors” or “Programmed Death Ligand 1 Inhibitors” or “PD-L1 Inhibitor” or “PD L1 Inhibitor” or “PD-1-PD-L1 Blockade” or “Blockade, PD-1-PD-L1” or “PD 1 PD L1 Blockade” or Nivolumab or Pembrolizumab or Sintilimab or Camrelizumab or Tislelizu-mab or Toripalimab or Penpulimab or Atezolizumab or Durvalumab or Envafolimab or Ipilimumab or Tremelimumab) (Topic) |
| #2 | (((((liver OR hepatic OR hepatocellular OR hepato-cellular) AND (cancer* OR carcinom*OR neoplasm? OR maglinan* OR tumor* OR tumour*))) OR (HCC OR hepatoma？)) ) (Topic) |
| #3 | #1 and #2 |
| #4 | #3 and Article or Review Article (Document Types) and 2014 or 2019 or 2018 or 2017 or 2016 or 2015 or 2020 or 2021 or 2022 or 2023 or 2024 (Publication Years) and English (Languages) |
